# Facile Synthesis of Nitrogen Self-Doped Porous Carbon Derived from Cicada Shell via KOH Activation for Simultaneous Detection and Removal of Cu^2+^

**DOI:** 10.3390/molecules27144516

**Published:** 2022-07-15

**Authors:** Jin Zou, Jiawei Liu, Qi Yu, Yansha Gao, Shangxing Chen, Xigen Huang, Dongnan Hu, Shuwu Liu, Limin Lu

**Affiliations:** 1 East China Woody Fragrance and Flavor Engineering Research Center of NF&GA, College of Forestry, JXAU, Nanchang 330045, China; 15797710413@163.com (J.Z.); qiyujxau@163.com (Q.Y.); 2 Key Laboratory of Chemical Utilization of Plant Resources of Nanchang, College of Chemistry and Materials, Jiangxi Agricultural University, Nanchang 330045, China; 15180018556@163.com (J.L.); gaoyansha@126.com (Y.G.); hxg.208@163.com (X.H.); suwu762846210@126.com (S.L.)

**Keywords:** cicada shell, natural N-doped biochar, heavy metal ion, electrochemical detection, adsorption

## Abstract

Sensitive detection and efficient removal of heavy metal ions with high toxicity and mobility are of great importance for environmental monitoring and control. Although several kinds of functional materials have been reported for this purpose, their preparation processes are complicated. Herein, nitrogen self-doped activated porous biochar (NAC) was synthesized in a facile process via an activation–carbonization strategy from cicada shell rich in chitin, and subsequently employed as an effective functional material for the simultaneous determination and removal of Cu^2+^ from aqueous media. With its unique porous structure and abundant oxygen-containing functional groups, along with the presence of heteroatoms, NAC exhibits high sensitivity for the electrochemical sensing of Cu^2+^ in concentrations ranging from 0.001 to 1000 μg·L^−1^, with a low detection limit of 0.3 ng·L^−1^. Additionally, NAC presents an excellent removal efficiency of over 78%. The maximum adsorption capacity is estimated at 110.4 mg/g. These excellent performances demonstrate that NAC could serve as an efficient platform for the detection and removal of Cu^2+^ in real environmental areas.

## 1. Introduction

It is well known that heavy metal ions (HMIs) can be accumulated in living organisms and bind to biological ligands containing N, S, and O, causing serious health problems [1]. Copper ion (Cu^2+^), as an indispensable element present in the human body, is vital to the normal functioning and metabolic processes of human organs [2]. However, higher or lower concentrations can disrupt the balance of Cu in the body, leading to several health problems, like neurodegenerative disease and Wilson’s disease [3]. Therefore, sensitive detection and effective separation strategies for Cu^2+^ in wastewater are urgently needed. Currently, some materials, such as COF, SnS-Bi_2_O_3_ and UiO-66-decorated three-dimensional microporous carbon [4,5,6], have been reported for the simultaneous detection and adsorption of HMIs. These materials display high sensitivity and effective removal for HMIs, but their synthesis methods are slightly complicated. In addition, the utilization of organic solvents in the synthesis process is counterproductive to the purpose of environmental protection. In this context, it is a challenging task to design novel bifunctional materials using simple and inexpensive synthesis routes.

Recently, biochar has been attracting immense interest for potential applications in the energy, adsorption and sensing fields due to its low cost, rapid regeneration, and green preparation methods [7,8]. On one hand, biochar typically has good electrical conductivity and a highly functionalized surface that contains peripheral groups that are able to chelate metal ions, providing a solid basis for HMIs sensing [9]. On the other hand, biochar is also regarded as an attractive adsorbent for HMIs owing to its large surface area, high degree of surface reactivity, and abundance of minerals. Moreover, biomass is a rich source of the elements C, N and O, and contains traces of Na, K, Fe, and Ca [10,11,12]. Therefore, without introducing additional nitrogen sources, directly carbonizing nitrogen-rich biomass can generate nitrogen-doped biochar (NBC), which is expected to be a promising candidate material for the simultaneous removal and detection of HMIs. However, currently, related research on biochar either focuses on the adsorption and removal of HMIs, or on the sensing of HMIs. The simultaneous detection and removal of HMIs using biochar has rarely been reported. 

In general, NBC is obtained from biomass materials by means of direct carbonization without the use of any chemical reagents during thermal processing. However, NBC prepared in this way usually manifests with low specific surface area, underdeveloped porosity, and poor catalytic activity, which greatly limits its application in the treatment of HMIs [13]. To enhance the performance of biochar, KOH is usually employed as an activator to prepare activated biochar with a well-developed pore structure, abundant active sites, and large specific surface area. During the carbonization process, KOH can be decomposed, together with carbon precursors, into a large amount of gaseous CO, CO_2_ and H_2_ at high temperature. KOH not only acts as a catalyst in the dehydration process of carbon precursors [14], but the redox reactions between various potassium compounds also corrode the carbon skeleton to modulate the pore structure [15]. 

On the basis of the above considerations, in this work, N-doped activated biochar (NAC) was prepared using a simple one-pot activation–carbonization strategy, and cicada shell (CS) rich in chitin, protein and amino acids was selected as the raw material. With the help of KOH, the obtained NAC exhibits a large effective surface area, a good microporous structure, and high heteroatom functionalization, which are potentially promising for the detection and removal of Cu^2+^. The electrochemical performance of the NAC is studied using the DPASV method, and the electrochemical sensor based on NAC-modified electrode presents good stability, high sensitivity, and excellent selectivity. The interaction between the doped nitrogen and Cu^2+^ is examined on the basis of adsorption experiments and X-ray photoelectron spectroscopy (XPS). The prepared NAC shows strong adsorption for Cu^2+^, with a maximum adsorption capacity of 110.4 mg·g^−1^.

## 2. Results and Discussion

### 2.1. Characterization of NAC

**Figure 1:**
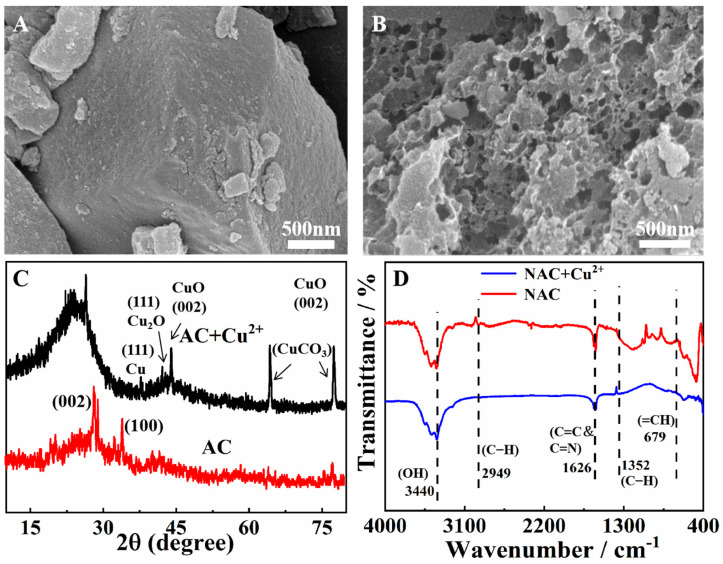
(**A**,**B**) SEM images of (**A**) NBC and (**B**) NAC. (**C**) XRD diffraction patterns and (**D**) FTIR spectra of NAC before and after adsorption.

The morphological characteristics of NBC and NAC were observed by scanning electron microscopy (SEM). As shown in Figure 1A, NBC obtained by direct carbonization exhibits large irregular blocks. However, with the use of KOH as activator, NAC shows a developed porous structure with a rougher surface (Figure 1B). During the hydrothermal carbonization process, chitin in CS was deacetylated to produce chitosan, which was then closely mixed with KOH. Subsequently, the mixture of chitosan and KOH was annealed at 800 °C in Ar atmosphere, and a large number of pores were produced. These porous structures expose abundant active sites, enhancing the mass transfer properties and providing natural channels for the diffusion of electrolytes [16]. 

The structural features of the as-prepared samples were identified on the basis of X-ray diffraction (XRD) patterns (Figure 1C). As can be seen, the characteristic peaks of NAC located at 2θ of 26.0° and 31.8° correspond to the (002) and (100) planes, respectively. The broad diffraction peaks indicate a highly disordered carbon species [17]. The Fourier transform infrared spectroscopy (FTIR) results of the NAC sample are presented in Figure 1D. The characteristic peaks appearing at 3440 and 2949 cm^−1^ represent the O-H and C-H stretching vibrations, respectively. The peak at 1352 cm^−1^ can be attributed to the C-H bending vibration, and the peak at 1626 cm^−1^ can be attributed to C=C or C=N stretching vibration. At the same time, a weak band at around 679 cm^−1^ indicates the =CH aromatic group [18]. These results imply that the NAC material possesses a certain level of N-containing and O-containing hydrophilic functional groups.

**Figure 2:**
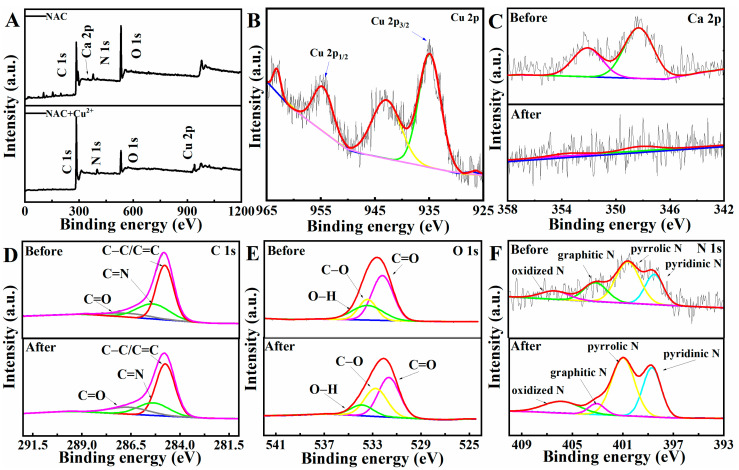
XPS diffraction patterns for NAC before and after adsorption: full scan (**A**), high-resolution Cu 2p spectrum (**B**), high-resolution Ca 2p spectrum (**C**), high-resolution C 1s spectrum (**D**), high-resolution O 1s spectrum (**E**), and high-resolution N 1s spectrum (**F**).

The surface analyses of the NAC were obtained on the basis of XPS measurements. With the survey scanning spectra (Figure 2A), the presence of carbon, nitrogen, and oxygen in NAC is displayed. The C1s peaks (Figure 2D) at 284.8 eV, 285.5 eV, and 287.1 eV correspond to the sp^2^ bonded carbon atoms (C-C/C=C), C=N, and alkyl and ester groups, respectively [19]. For the O 1s spectrum (Figure 2E), there are three peaks located at 531.05 eV (C=O), 533.1 eV (C-O), and 534.1 eV (O-H). These oxygen-containing functional groups are expected to contribute to Cu^2+^ adsorption [20]. The N 1s spectra of NAC (Figure 2F) are resolved as four peaks at 401.2 eV (graphitic-N), 399.9 eV (pyrrolic-N), and 398.3 eV (pyridinic-N), and 402.9 eV (oxidized N), respectively [21]. The pyridine N and pyridine N account for a large proportion of N species, which is conducive to providing active sites and promoting the adsorption of Cu^2+^ on nitrogen-doped porous carbon [22].

### 2.2. Electrochemical Detection of Cu^2+^ Using NAC-Modified Electrode

#### 2.2.1. Electrochemical Characterization of NAC/GCE

**Figure 3:**
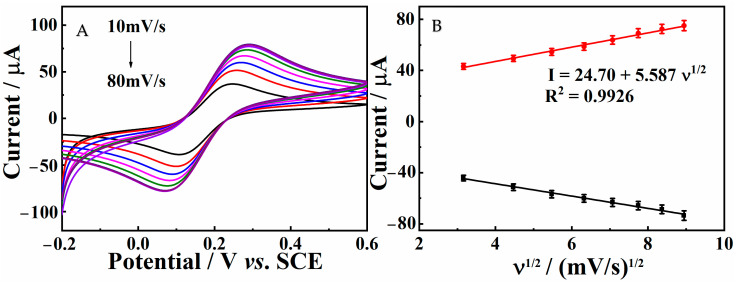
(**A**) CV curves of NAC/GCE at different scan rates; (**B**) linear relationship between the peaks current I_p_ and *v*^1/2^.

The electrochemically effective surface area (Aeff) of NAC/GCE was measured by performing cyclic voltammetry in a solution containing 5 mM [Fe(CN)_6_]^3^^−/4^^−^ and 0.1 M KCl at different scan rates (Figure 3A). Pursuant to the Randles–Sevcik principle [23], the Aeff values can be calculated on the basis of electrochemical measurements:

I_p_ = 2.69 × 10^5^ n^3/2^ A_eff_ D_0_^1/2^ C *v*^1/2^ (1)

where I_p_ refers to anode peak current (A), A represents the effective surface area of the electrode, D_0_ (0.76 × 10^−5^ cm^2^/s) and n (=1) are the diffusion coefficient and the number of electrons transferred, respectively. ν is the scan rate (V·s^−1^), and C_0_ indicates the concentration of the basal solution. Based on the anode peak standard equation of I = 5.587 *v*^1/2^ + 24.70 (R^2^ = 0.9926) (shown in Figure 3B), the effective surface area of NAC is estimated to be 0.0489 cm^2^. The value obtained suggests that NAC can provide a large effective surface area for electrochemical reactions, and then enhance the sensing signal.

#### 2.2.2. Electrochemical Measurements

**Figure 4:**
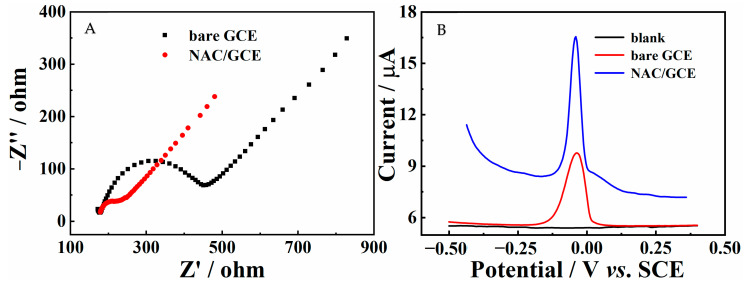
(**A**) Electrochemical impedance spectroscopy of bare GCE and NAC/GCE; (**B**) DPASV of 100 μg·L^−^^1^ Cu^2+^ at the bare GCE and NAC/GCE in 0.1 mol·L^−1^ acetate buffer solution (pH = 4.5).

Electrochemical impedance spectroscopy (EIS) was employed to investigate the interfacial charge transfer properties of bare GCE and NAC/GCE. The semi-circular arc at high frequency is equal to the charge transfer resistance (R_et_) of the electron transfer process, and the linear part at lower frequencies represents the Warburg impedance (Z_w_) of the diffusion process. Figure 4A depicts the EIS plots of bare GCE and NAC/GCE, and the inset shows the suitable electrical equivalent circuit. As can be observed, bare GCE presents a small semicircle with a Ret value of about 450 Ω. Compared with the bare GCE, the R_et_ value of NAC/GCE exhibits a significant decrease (52 Ω). This result suggests that NAC could provide a good electron pathway between the electrode and electrolyte, promoting the electron transfer and mass exchange of electroactive species on the film surface.

Figure 4B illustrates the electrochemical behaviors of GCE and NAC/GCE in NaAc-HAc (pH 5.0) against 100 μg·L^−1^ Cu^2+^. It can be seen that no stripping peak can be observed in a blank solution on bare GCE, suggesting that the electrode itself has no electrochemical activity under the measured conditions. However, when 100 μg·L^−1^ Cu^2+^ is added to the buffer solution, an obvious stripping peak can be observed at −0.132 V. Compared with bare GCE, a higher dissolution peak current can be observed for NAC/GCE, which is about 2-fold higher than that for bare GCE. This significant improvement may be caused by the good electronic conductivity and large surface area of the NAC. In addition, the increase in nitrogen content and oxygen-containing groups on the surface of the NAC promotes its interaction with Cu^2+^. These results indicate that NAC is a promising electrochemical material for fabricating Cu^2+^ sensors. 

#### 2.2.3. Optimization of Experimental Conditions

To obtain the best response of the developed sensor for the detection of Cu^2+^, a series of condition parameters were optimized, including supporting electrolyte, buffer pH, deposition potential, and deposition time.

**Figure 5:**
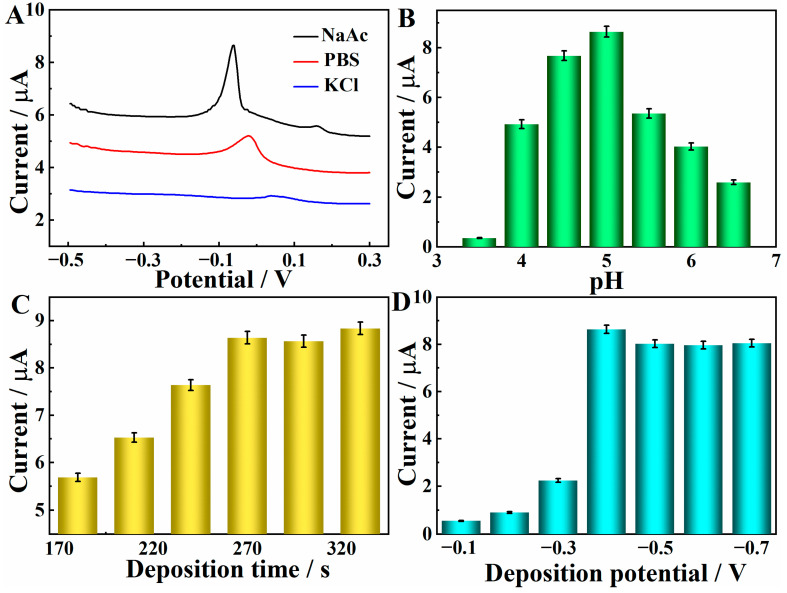
Optimization of the experimental conditions. Influence of (**A**) supporting electrolyte, (**B**) pH value of the detection solution, (**C**) deposition time, and (**D**) deposition potential on the stripping peak current of Cu^2+^ on the NAC/GCE.

Since the supporting solution has a significant influence on the performance of the modified electrode, herein, 0.6 M KCl solution (pH 5), 0.1 M phosphate buffer solution (PBS, pH 5), and 0.1 M HAc-NaAc buffer solution (ABS, pH 5) were chosen to perform measurements. The DPASV response of Cu^2+^ on NAC/GC in the three electrolyte solutions is depicted in Figure 5A. As shown, the peak signal of Cu^2+^ can hardly be seen in the 0.6 M KCl solution, while an obvious current response is seen in the 0.1 M PBS solution. Interestingly, in the 0.1 M ABS solution, the dissolution peak current intensity is significantly enhanced compared with that in PBS. Therefore, ABS was used as the dissolution electrolyte in the subsequent electrolytic analysis. 

The effect of various pH values (3.5–6.5) on the anodic peak of Cu^2+^ at NAC/GCE was studied. As can be observed in Figure 5B, the peak current of Cu^2+^ increased gradually as the buffer pH was increased from 3.5 to 5.0. At lower pH, the protonation of hydrophilic groups decreased the absorption of Cu^2+^. The sharp decrease in peak current at higher pH values is due to the hydrolysis of Cu^2+^. The maximum value was observed at a pH of 5.0. Accordingly, pH 5.0 was chosen for subsequent measurements.

The effect of the deposition time on the stripping responses of Cu^2+^ was explored from 180s to 330 s (Figure 5C). It was observed that the Cu^2+^ response increased with the extension of deposition time from 180 to 270 s, which can mainly be attributed to the increased amount of Cu^2+^ on the surface of the modified electrode. When the deposition time was increased to over 270 s, the stripping signal increased slightly or even leveled off, indicating the saturated binding of Cu^2+^ onto the electrode surface. Hence, the deposition time of 270 s was employed to perform the electrochemical measurements.

The effect of different deposition potentials (from −0.1 to −0.7 V) on Cu^2+^ response was further investigated (Figure 5D). The peak current of Cu^2+^ exhibited an increasing trend when the deposition potential was varied from −0.1 V to −0.4 V. With the negative shift of deposition potential, the stripping peak currents remained almost constant, which can be attributed to the competitive generation of H_2_, resulting in a decrease in the deposition of metal ions on the electrode surface [24]. As a result, −0.4 V was chosen for subsequent experiments. 

#### 2.2.4. DPASV Detection of Cu^2+^ at NAC/GCE

**Figure 6:**
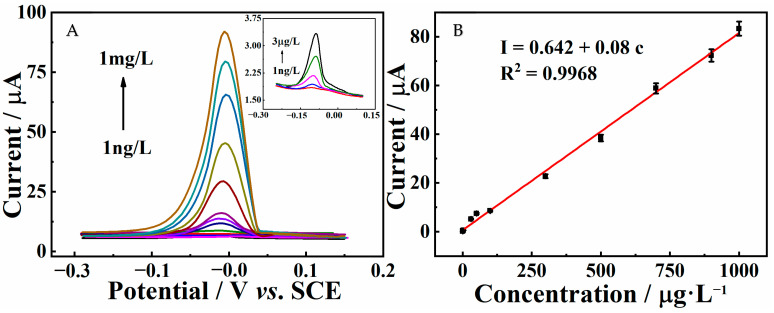
(**A**) DPASV of NAC/GCE in 0.1 M ABS (pH = 5.0) containing different concentrations of Cu^2+^; (**B**) the relationship between the peak current and the concentration of Cu^2+^ (detection range of 0.001~1000 μg·L^−^^1^).

Under the optimized conditions, the electrochemical performance toward Cu^2+^ at NAC/GCE was studied using DPASV. As shown in Figure 6A, Cu^2+^ displays a distinct peak at −0.057 V, and the peak current increases linearly when Cu^2+^ concentration is changed from 0.001 to 1000 μg·L^−1^. The regression equation is I_p_ (μA) = 0.08c + 0.642 (R^2^ = 0.9968) with a limit of detection (LOD) of 0.3 ng·L^−1^ (LOD = 3 SD/S), which is well below the drinking water guideline values of 1300 μg·L^−1^ established by the World Health Organization. Table 1 presents a comparison of the performance with other other substrates reported in the literature for Cu^2+^ detection, from which it can be found that the fabricated NAC/GCE possesses a lower LOD and a wider linear range. 

#### 2.2.5. Repeatability, Reproducibility, Stability, and Selectivity Measurements

**Figure 7:**
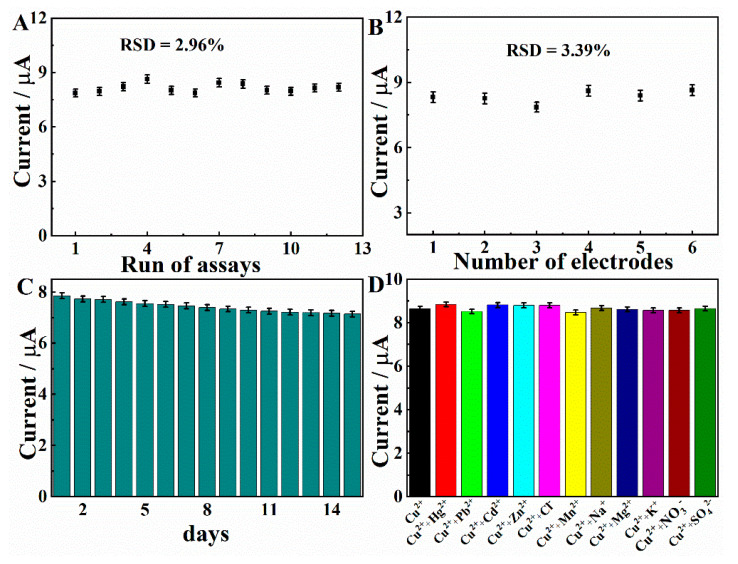
(**A**) Repeatability measurements for one NAC/GCE; (**B**) reproducibility measurements at seven independent NAC/GCEs; (**C**) stability measurements at NAC/GCE within 15 days; (**D**) selectivity of NAC/GCE. Deposition potential: −0.4 V. Deposition time: 270 s. Supporting electrolyte: 0.1 M acetate buffer (pH 5.0).

The repeatability of NAC/GCE toward 100 μg·L^−1^ Cu^2+^ was investigated using one modified electrode (Figure 7A). After 12 consecutive measurements, the relative standard deviation (RSD) was computed to be 2.96%, indicating the acceptable repeatability of the proposed sensor. The reproducibility of the NAC/GCE was also confirmed by detecting 100 μg·L^−1^ Cu^2+^ at seven individual electrodes. As shown in Figure 7B, the current response remained almost constant, and the RSD was calculated to be 3.39%, suggesting that the NAC/GCE has excellent reproducibility. 

The stability of the NAC/GCE was measured over 15 days by daily monitoring of the current response of 100 μg·L^−1^ Cu^2+^. As shown in Figure 7C, the current response retained 90.84% of the original current response after being stored for 15 days, indicating the excellent stability of the proposed sensor. 

The anti-interference ability of NAC/GCE was analyzed by adding different interference ions during the determination of 100 μg·L^−1^ Cu^2+^ (Figure 7D). The results show that 50-fold concentrations of Hg^2+^, Pb^2+^, Cd^2+^, Zn^2+^, Cl^−^, Mn^2+^, Na^+^, Mg^2+^, K^+^, NO^−^ and SO_4_^2^^−^ caused no obvious changes in the detection signal of Cu^2+^ (the peak current signal change was less than 5%), confirming that the proposed NAC/GCE has satisfactory anti-interference ability for the determination of Cu^2+^. 

#### 2.2.6. Recovery Study

To verify the practicality of NAC/GCE when analyzing real samples, paddy water from Jiangxi Agricultural University was used as real samples for quantitative analysis. The paddy water sample was first treated using a 0.22 μm filter membrane to settle the impurities, followed by dilution with 0.1 M ABS. The recovery study was performed by adding different concentrations of Cu^2+^ standard solutions into the paddy water samples. As shown in Table 2, it can be clearly seen that the obtained recovery was in the range of 99.13–102.0%, and the RSD value was less than 5%, indicating that the NAC/GCE is feasible for the detection of Cu^2+^ in real samples.

### 2.3. The Adsorption Studies of Cu^2+^ Using NAC

#### 2.3.1. Effect of Adsorption Conditions

**Figure 8:**
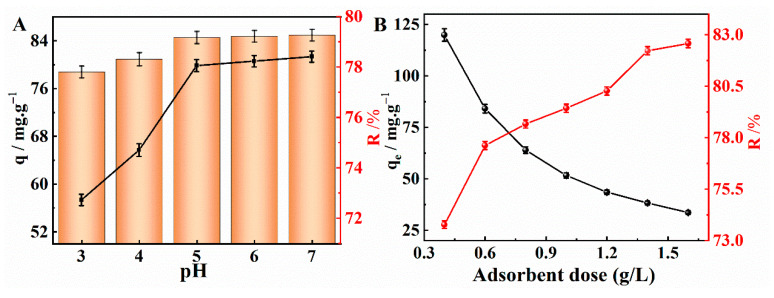
Effect of (**A**) pH and (**B**) adsorbent dose for Cu^2+^ adsorption on NAC.

To investigate the effects of pH on adsorption, experiments were conducted with pH values adjusted to within the range of 3.0–7.0 at 25 °C for 180 min using 0.6 g/L NAC and 65 mg/L Cu^2+^ solutions. As shown in Figure 8A, the removal efficiency of Cu^2+^ increases with increasing pH due to the protonation to deprotonation effect. At lower values of pH, the adsorption of Cu^2+^ is interfered with by excess H^+^, which competes with Cu^2+^ for adsorption sites. In addition, as the pH value changes, the surface electric charge of NAC and the type of Cu^2+^ change, and the electrostatic attraction between them is strengthened. When the pH value exceeds 6.0, the increased number of OH^−^ ions in the solution promotes Cu^2+^ precipitation in hydroxide form, which is adsorbed onto the porous material or container wall by means of physical interaction [29]. To avoid this precipitation, an initial pH of 5.0 is selected as the optimal condition for Cu^2+^ adsorption.

The effect of NAC dosage on the removal efficiency of Cu^2+^ was investigated (Figure 8B). Different dosages (0.4, 0.6, 0.8, 1.0, 1.2, 1.4 and 1.6 g·L^−1^) of NAC samples were used for the adsorption experiment in the presence of 65 mg·L^−1^ Cu^2+^ solution agitated at a rate of 250 rpm. When the adsorbent dosage was increased from 0.4 to 1.6 g·L^−1^, higher amounts of NAC provided more sorption sites, favoring the adsorption of Cu^2+^. When the dosage of adsorbent exceeded 0.6 g·L^−1^, the removal percentage increased slowly, and the adsorption capacity decreased due to the aggregation of NAC particles. In consideration of cost and effectiveness, 0.6 g·L^−1^ was selected as the optimal adsorbent dosage.

#### 2.3.2. Kinetic Study

**Figure 9:**
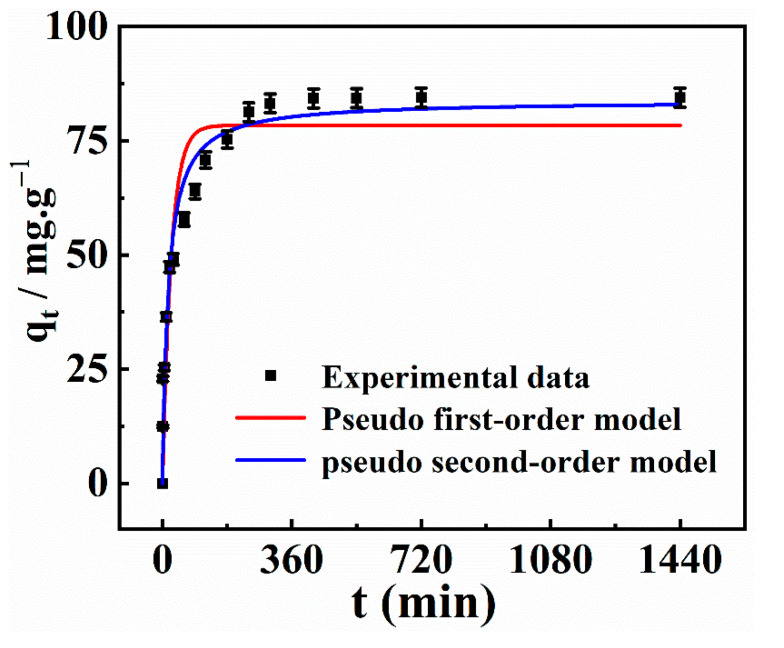
Influences of the uptake time and adsorption kinetics of Cu^2+^ onto NAC.

The influence of adsorption time (0–24 h) on Cu^2+^ adsorption is shown in Figure 9. The adsorption capacity of Cu^2+^ on NAC displays a sharp increase within five hours, followed by equilibrium being attained in about six hours. The faster first stage can be interpreted by the fact that the abundant binding sites enhance the contact between the adsorbent and adsorbate, with the reaction mainly occurring on the external biochar surface [30]. Meanwhile, with increasing time, the adsorption sites on NAC surface become saturated, and the effective adsorption sites are occupied by Cu^2+^.

All kinetics data were analyzed to fit the pseudo-first-order and pseudo-second-order kinetics models, and the expression formulas are given as follows [31]:

(2) $$q_{t}=q_{e}\left(1-e^{-k_{1}t}\right)$$

(3) $$q_{t}=q_{e}^{2}k_{2}t/\left(1+q_{e}k_{2}t\right)$$

where *q_t_* and *q_e_* are the adsorption capacity at time (*t*) and the adsorption equilibrium, respectively (mg/g). *k*_1_ (min^−1^) and *k*_2_ (g/mg·min) are the equilibrium kinetics models. The fitting plots between *t/q_t_* and *t*, as well as their straight-line fitting by linear regression analysis, are shown in Figure 9. The associated kinetic parameters determined using the two models are presented in Table 3. The R^2^ value (0.9467) for the second-order kinetics model is much higher than that of pseudo-first-order model (0.8939), demonstrating that the pseudo-second-order model provides a superior fit to that provided by the pseudo-first-order model for Cu^2+^. This means that for the pseudo-second-order model, the *q*_calc_ becomes more proximate to the experimentally measured *q*_exp_. The applicability of the pseudo-second-order kinetics model shows that the chemical adsorption process is the key control step in the process of Cu^2+^ adsorption on NAC.

#### 2.3.3. Adsorption Isotherms

**Figure 10:**
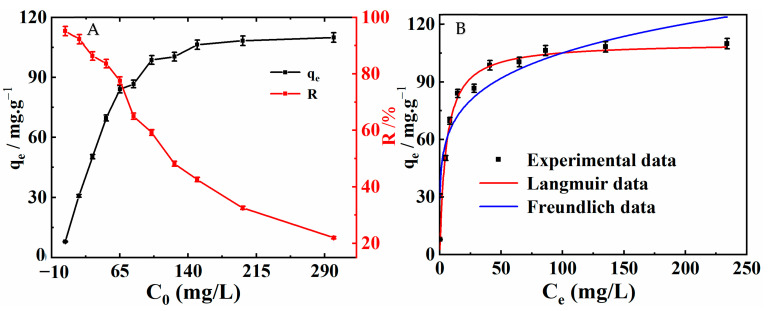
(**A**) Effect of initial Cu^2+^ concentration on Cu^2+^ adsorption; (**B**) adsorption isotherms for Cu^2+^ on NAC.

The effect of initial Cu^2+^ concentration on the adsorption effect is shown in Figure 10A. With increasing solution concentration, the Cu^2+^ adsorption capacity increases rapidly at first, and then reaches a plateau, while the removal efficiency decreases from 95.16% to 21.99%. For a given amount of NAC, the number of active adsorption sites on the surface of the adsorbent is relatively stable. When the initial concentration of Cu^2+^ is low, the availability of an enormous amount of adsorption sites enhances the ready binding of Cu^2+^ on the NAC surface. When the initial concentration is higher than 65 mg/L, the active sites on the adsorbent surface are saturated or even depleted with increasing Cu^2+^ concentration, resulting in a tapering increase in adsorption capacity [32]. Considering removal efficiency as well as adsorption capacity, the ideal initial Cu^2+^ concentration is 65 mg/L for NAC. 

To better interpret the adsorption process, different adsorption isotherm models (the Langmuir model and the Freundlich model) are used to describe the adsorption isotherms. The Langmuir model assumes that adsorption takes place at homogeneous sites on a uniform surface and can be written as Equation (3) [33]:

(4) $$q_{e}=q_{m}C_{e}K_{L}/\left(1+C_{e}K_{L}\right)$$

The Freundlich model assumes that the adsorption process is a nonuniform adsorption of multiple layers, and can be given by the following equation [34]:

(5) qe=KfCe1n

where *q_e_* is the equilibrium adsorption amount of Cu^2+^ on NAC material (mg/g), *q_m_* is the highest adsorption amount (mg/g), *C_e_* is the equilibrium concentration of Cu^2+^ (mg/L), n is the adsorption intensity, and *K_L_* and *K_f_* are the Langmuir isotherm constant and Freundlich adsorption constant, respectively.

Figure 10B is a nonlinear fitting diagram of the isothermal adsorption models of Cu^2+^, and the isotherm study results are presented in Table 4. From the point of view of the nonlinear correlative coefficient (R^2^), the R^2^ of the Langmuir model is larger than that of the Freundlich model, which shows that the Langmuir model describes the adsorption behavior of Cu^2+^ well. An R^2^ of 0.9120 was obtained for the Langmuir model, implying that the adsorption of NAC for Cu^2+^ is favorable [35]. Additionally, the uptake ability index 1/n (0.1936) of the Freundlich model was between 0 and 1, which further confirms the favorable adsorption of Cu^2+^ onto NAC and proves that the sorption process is nonlinear [36]. The theoretical qmax value calculated by the Langmuir model was 110.4 mg/g, which is comparable with that of other reported carbon adsorbents (Table 5). These results suggest that NAC has great potential for Cu^2+^ removal from aqueous solutions.

#### 2.3.4. Adsorption Mechanism

Research into the adsorption mechanism is beneficial for understanding the adsorption process of Cu^2+^ onto NAC adsorbents. To further investigate the adsorption mechanism of Cu^2+^ removal, the XPS spectra of NAC and NAC-Cu were determined. As can be observed from the spectra of NAC-Cu (Figure 2A), the peak of the Ca element disappears, whereas the Cu element shows a clear peak, demonstrating that ion exchange has occurred between Ca^2+^ and Cu^2+^ during the adsorption process. For the C1s spectra in Figure 2D, the peak (π-π* transition) at 296.4 eV disappears after Cu^2+^ adsorption. Meanwhile, the characteristic peaks (533.2 and 533.6 eV) of the O 1s spectra shift to a different binding energy after Cu^2+^ adsorption, indicating that the O atoms in -OH and -COOH have formed complexes with Cu^2+^ [41,42] (Figure 2E). Furthermore, from Figure 2F, it can be seen that the contents of pyridinic-N and oxidized-N decrease after the adsorption of Cu^2+^, proving that the nitrogen-containing functional groups on the NAC have reacted with Cu^2+^.

To identify the surface functional groups responsible for Cu^2+^ adsorption, FTIR analyses of NAC and NAC-Cu were also performed (Figure 1D). Following Cu^2+^ adsorption, the intensity of O-H banding at 3440 cm^−1^ decreases sharply, suggesting that the hydroxyl groups may be involved in the adsorption. Notably, the peak around 1626 cm^−1^ shifts to lower wavenumbers after the uptake of Cu^2+^. This is probably due to the fact that the lone pair of electrons from N atoms were shared with metal cations, resulting in a decreased electron cloud density of N atoms [41]. 

Furthermore, the XRD spectra of NAC treated with Cu^2+^ were recorded (Figure 1C). As can be seen, the special diffraction peaks and crystal planes of the Cu^2+^ crystal structure appear in the XRD patterns after absorption, located at 2θ = 44.8°, 63.1° and 76.2°, respectively. Moreover, the characteristic peak located at 2θ = 33.2° is weakened and shifted to 35.6°, which is related to the fact that the Ca^2+^ (0.99 Å), with a larger radius in the NAC, was partly or completely replaced by Cu^2+^ (0.72 Å) with smaller radii. This result demonstrates that the mechanism of Cu^2+^ adsorption by NAC is via ion exchange with Ca^2+^ [43]. 

In adsorption studies, pH is the most important parameter, having a great effect on the surface charge, degree of ionization, and speciation of the metal ions in the solution [44]. In lower pH regions, the electrostatic repulsion between biochar and Cu^2+^ decreases, resulting in enhanced removal efficiency. Therefore, the electrostatic interaction between NAC and Cu^2+^ is one reason for its stable adsorption of Cu^2+^. In a word, the adsorption process of Cu^2+^ in solution by NAC is mainly controlled by the chemical adsorption process. Possible mechanisms of Cu^2+^ adsorption by NAC include surface complexation, co-precipitation, electrostatic interaction, and ion exchange.

## 3. Experimental Section

### 3.1. Materials

The CS were collected from the woods in the north area of Jiangxi Agricultural University. Acetic acid (HAc) and sodium acetate (NaAc) were supplied by Tianjin Fuchen Chemical Reagent Ltd. (Tianjin, China). KOH, HCl, HNO_3_ and NaOH were obtained from Tianjin Damao Chemical Reagent Factory (Tianjin, China). Standard Cu^2+^ solution (1000 µg/mL) was obtained from Methrom (Herisau, Switzerland). All chemicals were used without any purification. 

### 3.2. Instruments

The surface morphology was measured using scanning electron microscopy (SEM, SU-8220). Fourier transform infrared (FTIR) spectra (Nicolet 6700, Waltham, MA, USA) was used for the identification of functional groups on the surface of NAC samples. The mineral constituents of NAC before and after Cu^2+^ adsorption were detected on an X-ray diffractometer (Bruker XFlash-SDD-5010, Karlsruhe, Germany). X-ray photoelectron spectroscopy (XPS) was conducted on an ESCALAB250Xi (Thermo Scientific, Waltham, MA, USA) equipped with a monochromatic Al Kα X-ray source (hν = 1486.6 eV). The metal ion concentrations in the solutions before and after the adsorption tests were measured using an atomic absorption spectrophotometer (AAS, PinAAcle 900T, Waltham, MA, USA). All electrochemical studies were performed using a CHI760E (CH Instruments, Shanghai, China) using a three-electrode system with 0.1 M acetate buffer solution (NaAc-HAc) as an electrolyte, the modified or bare GCE electrode (Φ = 3 mm) as the working electrode, platinum wire as an auxiliary electrode, and saturated calomel electrode (SCE) as a reference electrode. Differential pulse anodic stripping voltammetry (DPASV) was applied to carry out Cu^2+^ detection under optimized experimental conditions. Electrochemical characterization of NAC/GCE was performed in 0.1 M KCl solution containing 1.0 mM K_3_[Fe(CN)_6_]/K_4_[Fe(CN)_6_].

### 3.3. Preparation of NAC

NAC was prepared using the chemical activation technique with CS as a raw material. KOH was used as the chemical activator, and the carbonization temperature was 800 °C. First, 3 g of cicada shell was firstly washed with water to remove impurities. After drying, cicada shells were ground into powder and sifted through a 500-mesh sieve, and then mixed with 3 M KOH at a mass ratio of 2/1. The mixture was poured into a stainless-steel autoclave and activated at 150 °C for 6 h. After being dried under vacuum at 80 °C for 12 h, the activated cicada shell powder was pyrolyzed at 800 °C with a heating rate of 2 °C/min) for 5 h in a tube furnace under an Ar atmosphere. Finally, the obtained product was washed repeatedly with HCl until neutral, and centrifugally dried to obtain NAC.

### 3.4. Adsorption Studies of Cu^2+^

The adsorption experiment was performed in a 50 mL centrifuge tube, resulting in 25 mL solutions being obtained with predesignated concentrations of Cu^2+^ (5–300 mg·L^−1^) and biochar (0.6 g·L^−1^). The pH was adjusted to the desired value using HNO_3_ or NaOH solution. The mixture was then shaken at 298 K with a magnetic stirrer for a specific time. After the adsorption, the mixture was filtered through a syringe filter, and the Cu^2+^ concentration was determined by ASS, which was then used for isotherm and kinetics model analyses. The adsorbed amount (*q*, mg·g^−1^) and metal removal efficiency (*R*, %) of Cu^2+^ were subsequently calculated according to Equations (5) and (6), respectively [45,46].

(6) $$q=\frac{\left(c_{0}-c_{t}\right)V}{m}$$

(7) $$R=\frac{c_{0}-c_{t}}{c_{0}}\times 100\%$$

where *q* (mg/g) represents the mass of the Cu^2+^ adsorbed by each gram of NAC; *C*_0_ (mg/L) and *C_e_* (mg/L) represent the initial Cu^2+^ concentration and the equilibrium concentrations of Cu^2+^, respectively; *V* (L) represents the Cu^2+^ volume; and *m* (g) is the dose of NAC absorbent.

### 3.5. Fabrication of Modified Electrodes

**Scheme 1:**
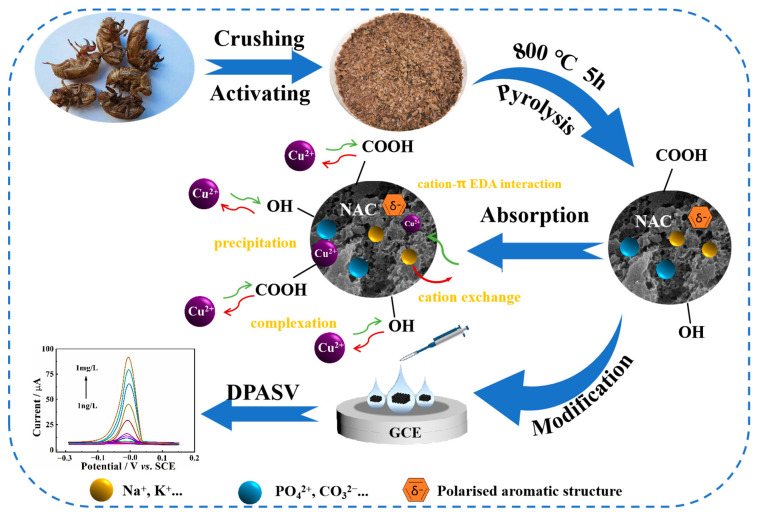
Schematic representation of the adsorption and electrochemical detection of Cu^2+^ using NAC.

In order to prepare the NAC-modified glass carbon electrode (GCE), NAC was ultrasonically dispersed in deionized water to obtain a homogeneous suspension (1 mg/mL). Before modification, the surface of the working electrode was properly polished using alumina slurry, and then rinsed thoroughly with ethanol and water. Subsequently, 5 μL NAC dispersion was coated on working electrodes for the fabrication of NAC/GCE. The detection and adsorption of Cu^2+^ are schematically shown in Scheme 1.

## 4. Conclusions

In this study, a porous N self-doped biochar was prepared using cicada shell subjected to chemical activation treatment for efficient Cu^2+^ detection and removal from aqueous solution. As a result of the well-organized microporous structure and the N, O-rich functional groups, the as-prepared NAC demonstrated high affinity and excellent adsorption ability toward Cu^2+^. The pseudo-second-order kinetics model and the Langmuir isotherm model best represented the adsorption data. Adsorption is believed to occur at the NAC surface via the mechanisms of co-precipitation, ion exchange, electrostatic interaction, and surface complexation. Meanwhile, NAC also demonstrates highly sensitive detection, with a low LOD for Cu^2+^ (0.3 ng·L^−1^). In summary, this work provides a green and facile approach to fabricating N-doped porous carbon material, which is promising for the detection and removal of Cu^2+^ in wastewater.

## Figures and Tables

**Table 1:** Comparison of the analytical performance of various reported electrochemical sensors for the sensing of Cu^2+^.

Electrode Substrate	Measurement Technique	Linear Range (μg·L^−^^1^)	LOD (μg·L^−^^1^)	References
COFBTLP-1/3D-KSC ^a^ electrode	DPSV	3.6–1200	1.2	[25]
SSA ^b^/MoS_2_/o-MWCNTs ^c^/GCE	DPASV	6.4–704	3.648	[26]
N, P-Ti_3_C_2_TxR ^d^/GCE	DPV	1.28–640	0.1152	[27]
AuNPs-GR ^e^/GCE	ASV	0.32–6.4	0.0018	[3]
N-CSs ^f^/MWCNTs ^g^-Nafion/GCE	DPASV	0.5–200	0.092	[28]
NAC/GCE	DPASV	0.001–1000	0.0003	This work

Note: ^a^ three-dimensional macroporous carbon; ^b^ 5-sulfosalicylic acid; ^c^ oxidized multi-walled carbon nanotubes; ^d^ N and P co-doped Ti_3_C_2_Tx MXenes nanoribbons; ^e^ graphene-based; ^f^ N-doped carbon spheres; ^g^ multi-walled carbon nanotubes.

**Table 2:** Recovery measurements of Cu^2+^ in paddy water samples using the constructed sensor (n = 3).

Sample	Added (μg·L^−^^1^)	Founded (μg·L^−^^1^)	Recovery (%)
1	0	-	-
2	0.5	0.51 ± 0.02	102.0
3	5.0	4.96 ± 0.07	99.13
4	50.0	49.77 ± 1.32	99.55
5	100.0	100.7 ± 2.40	100.7

**Table 3:** Adsorption kinetics parameters for adsorption of Cu^2+^ onto NAC.

	Pseudo-First-Order Model			Pseudo-Second-Order Model		
*q_e,exp_* (mg·g^−1^)	*k*_1_ (min^−1^)	R^2^	*q_e_* (mg·g^−1^)	*k*_2_ (g/mg·min)	R^2^	*q_e_* (mg·g^−1^)
84.41	2.485	0.8939	81.57	0.045	0.9467	83.81

**Table 4:** Adsorption parameters of Cu^2+^ on NAC material determined by Langmuir and Freundlich models.

Langmuir			Freundlich		
*q_e_* (mg/g)	*K*_1_ (L/mg)	R^2^	1/n	*K_L_* (mg/g)	R^2^
110.4	0.1974	0.9120	0.1936	43.11	0.8943

**Table 5:** Comparison of adsorption capacities with other forms of adsorbent material reported in the literature for Cu^2+^.

Used Adsorbents	Adsorption Capacity (mg/g)	Ref.
Porous adsorbent	37.50	[37]
Seaweed biochar	223.0	[38]
Activated carbons	23.90	[39]
Granular activated carbon	95.00	[40]
This work	110.4	This work

## Data Availability

The data presented in this study are available in the article.

## List of Abbreviations and Symbols

*Abbreviations*	
Acetic acid	HAc
Atomic absorption spectrophotometer	AAS
Acetate buffer solution	NaAc-HAc
Copper ion	Cu^2+^
Cicada shell	CS
Differential pulse anodic stripping voltammetry	DPASV
Electrochemically effective surface area	Aeff
Electrochemical impedance spectroscopy	EIS
Fourier transform infrared spectroscopy	FTIR
Graphene-based	GR
Glass carbon electrode	GCE
Heavy metal ions	HMIs
HAc-NaAc buffer solution	ABS
Limit of detection	LOD
Multi-walled carbon nanotubes	MWCNTs
Nitrogen-doped biochar	NBC
N-doped activated biochar	NAC
N and P co-doped Ti_3_C_2_Tx MXenes Nanoribbons	N, P-Ti_3_C_2_TxR
N-doped carbon spheres	N-CSs
Oxidized multi-walled carbon nanotubes	o-MWCNTs
Phosphate buffer solution	PBS
Sodium acetate	NaAc
Scanning electron microscopy	SEM
Saturated calomel electrode	SCE
Three-dimensional macroporous carbon	3D-KSC
X-ray photoelectron spectroscopy	XPS
X-ray diffraction	XRD
5-sulfosalicylic acid	SSA
*Symbols*	
Anode peak current	I_p_ (A)
Adsorption intensity	n
Cu^2+^ volume	*V* (L)
Freundlich adsorption constant	*K_f_* (mg/g)
Initial Cu^2+^ concentration	*C*_0_ (mg/L)
Langmuir isotherm constant	*K_L_* (L/g)
Nonlinear correlative coefficient	R^2^
Pseudo-first-order sorption rate constant	*k*_1_ (min^−^^1^)
Pseudo-second-order sorption rate constant	*k*_2_ (g/mg·min)
Relative standard deviation	RSD
Diffusion coefficient	*D*_0_ (0.76 × 10^−^^5^ cm^2^/s)
Number of electrons transferred	n (=1)
Scan rate	ν (V·s^−^^1^)
Concentration of the basal solution	*C*_0_ (mM)
Charge transfer resistance	R_et_ (Ω)
Warburg impedance	*Z_w_*
Time	*t* (min)
Equilibrium adsorption amount	*q_e_* (mg/g)
Adsorption capacity at time t	*q_t_* (mg/g)
Highest adsorption amount	*q_m_* (mg/g)
Equilibrium concentration of Cu^2+^	*C_e_* (mg/L)
Dose of NAC absorbent	*m* (g)

## References

[1] Atangana E., Oberholster P.J. (2020). Oberholster, Modifed Biopolymer (Chitin-Chitosan Derivatives) for the Removal of Heavy Metals in Poultry Wastewater. J. Polym. Environ..

[2] Zhang Y.X., Qiu G.F., Wang R.M., Guo Y., Guo F.H., Wu J.J. (2021). Preparation of Bamboo-Based Hierarchical Porous Carbon Modulated by FeCl_3_ towards Efficient Copper Adsorption. Molecules.

[3] Wang S., Wang Y., Zhou L., Li J., Wang S., Liu H. (2014). Fabrication of an effective electrochemical platform based on graphene and AuNPs for high sensitive detection of trace Cu^2+^. Electrochim. Acta.

[4] Han J., Pei L., Du Y., Zhu Y. (2022). Tripolycyanamide-2,4,6-triformyl pyrogallol covalent organic frameworks with many coordination sites for detection and removal of heavy metal ions. J. Ind. Eng. Chem..

[5] Jin W., Fu Y., Hu M., Wang S., Liu Z. (2020). Highly efficient SnS-decorated Bi_2_O_3_ nanosheets for simultaneous electrochemical detection and removal of Cd(II) and Pb(II). J. Electroanal. Chem..

[6] Pei L., Yang H., Chen S., Wang L. (2022). UiO-66-NHC(S)NHMe/Three-Dimensional Macroporous Carbon for Removal and Electrochemical Detection of Cd^2+^, Pb^2+^, Cu^2+^, and Hg^2+^. Ind. Eng. Chem. Res..

[7] Rahman M.Z., Edvinsson T., Kwong P.C. (2020). Biochar for electrochemical applications. Curr. Opin. Green Sustain. Chem..

[8] Tian R., Li C., Xie S., You F., Cao Z., Xu Z., Yu G., Wang Y. (2019). Preparation of biochar via pyrolysis at laboratory and pilot scales to remove antibiotics and immobilize heavy metals in livestock feces. J. Soils Sediments.

[9] Zhu X., Liu B., Chen S., Wu L., Yang J., Liang S., Xiao K., Hu J., Hou H. (2020). Ultrasensitive and Simultaneous Electrochemical Determination of Pb^2+^ and Cd^2+^ Based on Biomass Derived Lotus Root-Like Hierarchical Porous Carbon/Bismuth Composite. J. Electrochem. Soc..

[10] Lin G., Ma R., Zhou Y., Liu Q., Dong X., Wang J. (2018). KOH activation of biomass-derived nitrogen-doped carbons for supercapacitor and electrocatalytic oxygen reduction. Electrochim. Acta..

[11] Wan W., Wang Q., Zhang L., Liang H.-W., Chen P., Yu S.-H. (2016). N-, P- and Fe-tridoped nanoporous carbon derived from plant biomass: An excellent oxygen reduction electrocatalyst for zinc-air batteries. J. Mater. A.

[12] Liu X., Zhou Y., Zhou W., Li L., Huang S., Chen S. (2015). Biomass-derived nitrogen self-doped porous carbon as effective metal-free catalysts for oxygen reduction reaction. Nanoscale.

[13] Long C., Jiang L., Wu X., Jiang Y., Yang D., Wang C., Wei T., Fan Z. (2015). Facile synthesis of functionalized porous carbon with three-dimensional interconnected pore structure for high volumetric performance supercapacitors. Carbon.

[14] Kim Y.K., Park J.H., Lee J.W. (2018). Facile nano-templated CO_2_ conversion into highly interconnected hierarchical porous carbon for high-performance supercapacitor electrodes. Carbon.

[15] Romanos J., Beckner M., Rash T., Firlej L., Kuchta B., Yu P., Suppes G., Wexler C., Pfeifer P. (2012). Nanospace engineering of KOH activated carbon. Nanotechnology.

[16] Gao F., Qu J., Zhao Z., Wang Z., Qiu J. (2016). Nitrogen-doped activated carbon derived from prawn shells for high-performance supercapacitors. Electrochim. Aata..

[17] Bao D., Li Z., Tang R., Wan C., Liu X. (2021). Metal-modified sludge-based biochar enhance catalytic capacity: Characteristics and mechanism. J. Environ. Manag..

[18] Inyang M., Gao B., Pullammanappallil P., Ding W., Zimmerman A.R. (2010). Biochar from anaerobically digested sugarcane bagasse. Bioresource Technol..

[19] Mondal A.K., Kretschmer K., Zhao Y., Liu H., Fan H., Wang G. (2017). Naturally nitrogen doped porous carbon derived from waste shrimp shells for high-performance lithium ion batteries and supercapacitors. Microporous Mesoporous Mater..

[20] Hulicova-Jurcakova D., Seredych M., Lu G.Q., Bandosz T.J. (2009). Combined Effect of Nitrogen- and Oxygen-Containing Functional Groups of Microporous Activated Carbon on its Electrochemical Performance in Supercapacitors. Adv. Funct. Mater..

[21] Wu F., Gao J., Zhai X., Xie M., Sun Y., Kang H., Tian Q., Qiu H. (2019). Hierarchical porous carbon microrods derived from albizia flowers for high performance supercapacitors. Carbon.

[22] Cao Y., Huang J., Li Y., Qiu S., Liu J., Khasanov A., Khan M.A., Young D.P., Peng F., Cao D. (2016). One-pot melamine derived nitrogen doped magnetic carbon nanoadsorbents with enhanced chromium removal. Carbon.

[23] Zhu D., Chu M.Y., Xin J.J., Wang X.M., O’Halloran K.P., Ma H.Y., Pang H.J., Tan L.C., Yang G.X. (2021). Hierarchical and hollow boron/nitrogen co-doped yolk-shell mesoporous carbon nanospheres attached to reduced graphene oxide with high sensing performance for the simultaneous detection of xanthine and guanosine. Sens. Actuators B.

[24] Lu M.X., Deng Y.J., Yi L., Lv J.P., Li T.B., Xu J., Chen S.W., Wang J.Y. (2018). Graphene aerogelmetal-organic framework-based electrochemical method for simultaneous detection of multiple heavy metal ions. Anal. Chem..

[25] Han J., Yu J., Guo Y., Wang L., Song Y. (2020). COFBTLP-1/three-dimensional macroporous carbon electrode for simultaneous electrochemical detection of Cd^2+^, Pb^2+^, Cu^2+^ and Hg^2+^. Sens. Actuators B.

[26] Wang S., Li J., Qiu Y., Zhuang X., Wu X., Jiang J. (2019). Facile synthesis of oxidized multi-walled carbon nanotubes functionalized with 5-sulfosalicylic acid/MoS_2_ nanosheets nanocomposites for electrochemical detection of copper ions. Appl. Surf. Sci..

[27] Xia Y., Zhao Y., Ai F., Yi Y., Liu T., Lin H., Zhu G. (2022). N and P co-doped MXenes nanoribbons for electrodeposition-free stripping analysis of Cu(II) and Hg(II). J. Hazard. Mater..

[28] Qin D., Mamat X., Li Y., Hu X., Cheng H., Hu G. (2020). A composite with botryoidal texture prepared from nitrogen-doped carbon spheres and carbon nanotubes for voltammetric sensing of copper(II). Microchem. J..

[29] Yang S., Hu J., Chen C., Shao D., Wang X. (2011). Mutual Effects of Pb(II) and Humic Acid Adsorption on Multiwalled Carbon Nanotubes/Polyacrylamide Composites from Aqueous Solutions. Environ. Sci. Technol..

[30] Pellera F., Giannis A., Kalderis D., Anastasiadou K., Stegmann R., Wang J., Gidarakos E. (2012). Adsorption of Cu(II) ions from aqueous solutions on biochars prepared from agricultural by-products. J. Environ. Manag..

[31] Ho Y.S., McKay G. (1999). Pseudo-second order model for sorption processes. Process Biochem..

[32] Amin M.T., Alazba A.A., Shafiq M. (2019). Application of biochar derived from date palm biomass for removal of lead and copper ions in a batch reactor: Kinetics and isotherm scrutiny. Chem. Phys. Lett..

[33] Foo K.Y., Hameed B.H. (2010). Insights into the modeling of adsorption isotherm systems. Chem. Eng. J..

[34] Zhou Q., Liao B., Lin L., Qiu W., Song Z. (2018). Adsorption of Cu(II) and Cd(II) from aqueous solutions by ferromanganese binary oxide-biochar composites. Sci. Total Environ..

[35] Cheng Q., Huang Q., Khan S., Liu Y., Liao Z., Li G., Ok Y.S. (2016). Adsorption of Cd by peanut husks and peanut husk biochar from aqueous solutions. Ecol. Eng..

[36] Wang C., Wang H. (2018). Pb(II) sorption from aqueous solution by novel biochar loaded with nano-particles. Chemosphere.

[37] Han J., Du Z., Zou W., Li H., Zhang C. (2015). In-situ improved phenol adsorption at ions-enrichment interface of porous adsorbent for simultaneous removal of copper ions and phenol. Chem. Eng. J..

[38] Katiyar R., Patel A.K., Nguyen T., Singhania R.R., Chen C., Dong C. (2021). Adsorption of copper (II) in aqueous solution using biochars derived from Ascophyllum nodosum seaweed. Bioresource Technol..

[39] Gu S., Hsieh C., Gandomi Y.A., Yang Z., Li L., Fu C., Juang R. (2019). Functionalization of activated carbons with magnetic Iron oxide nanoparticles for removal of copper ions from aqueous solution. J. Mol. Liq..

[40] Azizian S., Haerifar M., Bashiri H. (2009). Adsorption of methyl violet onto granular activated carbon: Equilibrium, kinetics and modeling. Chem. Eng. J..

[41] Guan D., Ren C., Wang J., Zhu Y., Zhu Z., Li W. (2018). Characterization of Lead Uptake by Nano-Sized Hydroxyapatite: A Molecular Scale Perspective. ACS Earth Space Chem..

[42] Yu W., Hu J., Yu Y., Ma D., Gong W., Qiu H., Hu Z., Gao H. (2021). Facile preparation of sulfonated biochar for highly efficient removal of toxic Pb(II) and Cd(II) from wastewater. Sci. Total Environ..

[43] Yu W., Lian F., Cui G., Liu Z. (2018). N-doping effectively enhances the adsorption capacity of biochar for heavy metal ions from aqueous solution. Chemosphere.

[44] Esvandi Z., Foroutan R., Mirjalili M., Sorial G.A., Ramavandi B. (2019). Physicochemical Behavior of Penaeuse semisulcatuse Chitin for Pb and Cd Removal from Aqueous Environment. J. Polym. Environ..

[45] Ding L., Luo X., Shao P., Yang J., Sun D. (2018). Thiol-Functionalized Zr-Based Metal-Organic Framework for Capture of Hg(II) through a Proton Exchange Reaction. ACS Sustain. Chem. Eng..

[46] Şenol Z.M., Şimşek S. (2022). Insights into Effective Adsorption of Lead ions from Aqueous Solutions by Using Chitosan-Bentonite Composite Beads. J. Polym. Environ..

